# The anti-malarial drug Mefloquine disrupts central autonomic and respiratory control in the working heart brainstem preparation of the rat

**DOI:** 10.1186/1423-0127-19-103

**Published:** 2012-12-15

**Authors:** Varinder K Lall, Mathias Dutschmann, Jim Deuchars, Susan A Deuchars

**Affiliations:** 1 School of Biomedical Sciences, University of Leeds, Leeds, UK

**Keywords:** Mefloquine, Malaria, Sympathetic, Connexin, Respiratory

## Abstract

**Background:**

Mefloquine is an anti-malarial drug that can have neurological side effects. This study examines how mefloquine (MF) influences central nervous control of autonomic and respiratory systems using the arterially perfused working heart brainstem preparation (WHBP) of the rat. Recordings of nerve activity were made from the thoracic sympathetic chain and phrenic nerve, while heart rate (HR) and perfusion pressure were also monitored in the arterially perfused, decerebrate, rat WHBP. MF was added to the perfusate at 1 μM to examine its effects on baseline parameters as well as baroreceptor and chemoreceptor reflexes.

**Results:**

MF caused a significant, atropine resistant, bradycardia and increased phrenic nerve discharge frequency. Chemoreceptor mediated sympathoexcitation (elicited by addition of 0.1 ml of 0.03% sodium cyanide to the aortic cannula) was significantly attenuated by the application of MF to the perfusate. Furthermore MF significantly decreased rate of return to resting HR following chemoreceptor induced bradycardia. An increase in respiratory frequency and attenuated respiratory-related sympathetic nerve discharge during chemoreceptor stimulation was also elicited with MF compared to control. However, MF did not significantly alter baroreceptor reflex sensitivity.

**Conclusions:**

These studies indicate that in the WHBP, MF causes profound alterations in autonomic and respiratory control. The possibility that these effects may be mediated through actions on connexin 36 containing gap junctions in central neurones controlling sympathetic nervous outflow is discussed.

## Background

Mefloquine (MF) is an anti-malarial drug which has been widely used for the treatment of malaria. Since MF has an elimination half life of between 14 to 41 days [1], it has been a common choice as a malaria prophylaxis drug. However, MF can clearly influence the central nervous system since patients taking MF as an anti-malarial report neuropsychiatric adverse reactions which include anxiety, strange and vivid dreams, hallucinations, psychosis [2,3].

In addition to neuropsychiatric effects, patients taking MF as an anti-malarial exhibited a significant bradycardia 6 days after administration [4]. They reported that the bradycardia observed may have been as a result of autonomic dysfunction rather than an effect on the heart itself since the PR-interval, QRS-interval and QTc intervals were not affected by this dose. Furthermore, severe postural orthostatic tachycardia syndrome including symptoms of palpitations and dizziness, loss of R-R interval variation, typical of autonomic dysfunction, were reported in a case study of a woman taking MF as an anti-malarial [5]. MF may have further consequences, as respiratory complications including dyspnoea and respiratory distress have been ascribed to MF in case studies of patients treated for malaria [6,7].

This study aims to explore the neural mechanisms of the significant cardio-respiratory side effects of MF malaria treatment. Exactly how MF causes the side effects in central autonomic circuits and on cardiovascular regulation is unknown, although one possible action of MF is through blockade of gap junctions (GJs) containing the GJ protein connexin 36 (Cx36) [8]. The brainstem and spinal cord contain several neuronal populations which display electrical coupling likely to be mediated by Cx36 containing GJs, including brainstem respiratory [9] and spinal sympathetic preganglionic neurones (SPNs) [10-12]. Since gap junction communication between neurones can be inhibited by anaesthetics [13], experiments were conducted in an anaesthetic free, arterially perfused working heart brainstem preparation (WHBP).

## Methods

### Surgical procedures

18 day old, male Wistar rats were deeply anaesthetised with halothane (3–5%) and once the animal failed to respond to noxious pinch to the tail or hind paw, the animal was bisected sub-diaphragmatically. The head and thorax were submerged in ice cold (5°C) modified artificial cerebrospinal fluid (aCSF, see below for composition) equilibrated with carbogen (95% O_2_, 5% CO_2_). The animal was decerebrated at the precollicular level and skinned. The dorsal surface of the brainstem was exposed by removal of the skull and cerebellum, the brainstem was superfused with modified aCSF. The phrenic and sympathetic nerves were isolated and the diaphragm, lungs and surrounding organs were removed. The descending aorta was isolated and the posterior thorax and spinal column were removed to mid thoracic level.

The preparation was transferred to a recording chamber and the descending aorta cannulated and perfused retrogradely with modified aCSF (pH 7.35 ± 0.05) at 32°C containing Ficoll 70 (Sigma-Aldrich), using a roller pump (Watson-Marlow). The perfusate was filtered with a nylon mesh to prevent blood clots and cellular debris from blocking the capillary beds. Perfusate was passed through two bubble traps which removed gas bubbles and reduced pulsations from the roller pump and heart. Aortic perfusion pressure was measured by a double lumen catheter connected to a pressure transducer. The perfusate was recycled after reoxygenation to limit loss of washed out nutrients such as amino acids. Bath temperature was maintained at 28°C using a heat chamber.

### Nerve recordings

To monitor central respiratory drive, recordings were made from the central end of the cut phrenic nerve using a glass suction electrode and the raw data recorded using Spike 2 (Cambridge Electronic Design, CED). Cardiac activity, recorded in the form of electrocardiography (ECG), returned within seconds and rhythmic contractions of respiratory muscles within a few minutes after the onset of reperfusion. Sympathetic nerve recordings were made from the lower thoracic sympathetic chain also using a glass suction electrode. This sympathetic activity exhibited respiratory modulation in all cases. HR was derived from ECG, recorded simultaneously with the phrenic and sympathetic nerve recordings.

### Solutions

Modified aCSF contained (in mM): NaCl, 125; NaHCO3, 24; KCl, 5; CaCl2, 2.5; MgSO4, 1.25; KH2PO4 1.25 and D glucose, 10. In addition 1.25%, Ficoll, (type 70; Sigma Aldrich, Gillingham, UK) was added to the perfusate. Stock solutions of atropine sulphate (1 mM in H_2_0, Sigma Aldrich), MF (1 Mm in dimethyl sulfoxide; Sigma Aldrich) were prepared and aliquots were frozen at −20°C. Prior to each experiment, aliquots were thawed and added to the perfusate (where stated).

### Autonomic reflexes

To test the effects of MF on specific reflex responses, the preparation was subject to manipulation of the cardiorespiratory system. Baroreceptors were stimulated by a transient increase in perfusion pressure (by increasing the speed of the roller pump to maximum rotation) and chemoreceptors were activated by injection of 0.1 ml of 0.03% sodium cyanide (NaCN) into the descending aorta (via a side arm port of the perfusion cannula).

### Analysis

Signals were recorded using glass suction electrodes attached to a head stage (Digitimer, NL100) and fed into a Neurolog amplifier (x 1000 amplification; Digitimer, NL900D). Signals were passed through a Humbug (Quest Scientific, Canada) to filter out mains noise at 50/60 Hz. Recordings were bandpass filtered between 50 Hz and 4 kHz. Recordings were digitised with a sampling frequency of 8 kHz and saved on computer using an interface (CED 1401, UK) to be analysed with Spike 2 software off line.

Sympathetic nerve discharge (SND) was rectified and integrated (∫SND) with a time constant of 100 ms. The difference in ∫SND from baseline to chemoreceptor/ baroreceptor stimulation was calculated in control and in MF using a custom written Spike2 script described previously [14]. ∫SND was measured 10 seconds before the autonomic reflex was elicited, during the response and after the autonomic reflex stimulation. At least three trials were elicited in each preparation during control, MF and washout and at least 5 minutes were allowed to elapse between each trial.

Phrenic nerve discharge (PND) was amplified and filtered then integrated and rectified (time constant of 100 ms). Stable periods of baseline PND, as recorded from Spike 2, were analysed off line to produce averages for Ti (total inspiration) duration, Te (total expiration) duration, Ttot (total inspiration and expiration) duration (seconds) and PND frequency (per minute), for at least 30 cycles per preparation. Activity was then analysed during chemoreceptor and baroreceptor stimulation. After such autonomic reflexes HR and PND were compared during the stimulus and for 5–10 respiratory cycles immediately following the stimulus. Three trials per preparation were averaged.

We also measured the amplitude of rectified and integrated ∫SND for the first 500 ms of the PND (start of the augmenting phase, considered early inspiration) and compared it to the ∫SND amplitude for the first 500 ms of silencing of the phrenic nerve (equivalent to post-inspiration as described by [14]). These two amplitudes were expressed as a ratio and compared in control and MF to give a measure of the degree of respiratory-related ∫SND in the two conditions. During chemoreceptor stimulation, the duration of Ti and Te was reduced considerably, thus respiratory-related ∫SND under these conditions was assessed by measurement of ∫SND during either total inspiration or expiration duration.

Unless stated, data are presented as group mean ± the standard error of the mean (S.E.M) and differences were considered significant at the 95% confidence limit. For each of the respective phases, significance was determined by paired student *t*-test (p< 0.05); “n” represents the number of preparations.

### Mefloquine addition

Once a stable preparation had been established and baseline autonomic reflexes had been conducted, 1 μM MF was added directly into the perfusate. Autonomic reflexes were elicited in the presence of MF and after washout of MF.

## Results

### Monitoring the preparation

Preparation viability was confirmed by the presence of rhythmic and ramping PND [15], arterial baroreceptor or peripheral chemoreceptor mediated bradycardia, and an increase in phrenic motor activity induced by injection of NaCN. Constant perfusion pressure during recordings from the WHBP allowed the brainstem to remain adequately oxygenated.

### Effects of MF on resting heart rate, sympathetic and phrenic nerve activity

**Figure 1:**
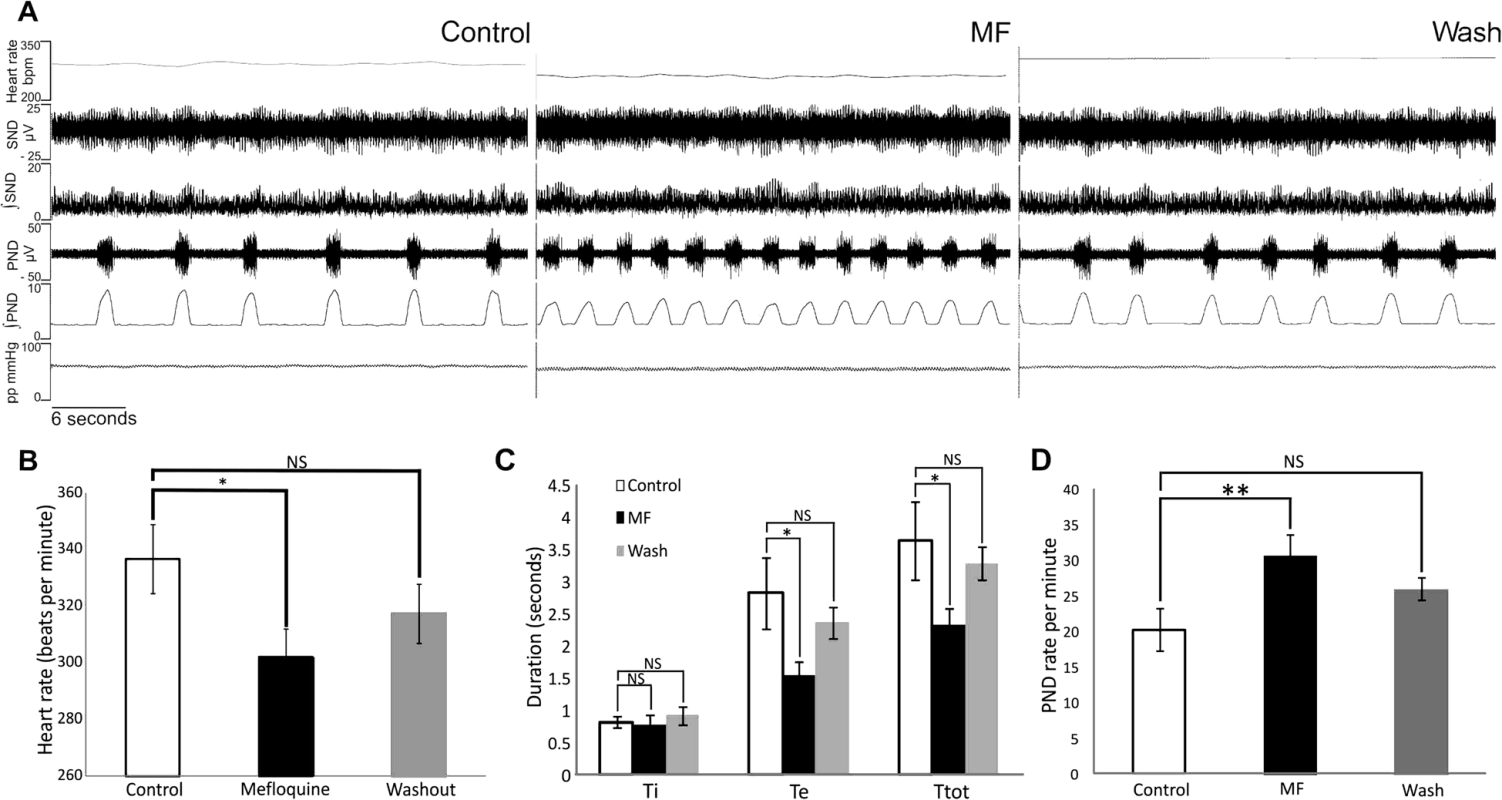
**Effects of MF on baseline heart rate, sympathetic nerve activity and phrenic nerve activity.** (**A**) Example, original traces showing baseline heart rate, sympathetic nerve and phrenic nerve activity during control, MF and washout. Heart rate significantly decreases (**B**) and PND rate significantly increases due to a significant reduction in the Te duration (seconds) (**C** and **D**) upon the application of MF to the perfusate, the effects are reversed after washout. Asterisks denote significance.

The average baseline HR was 336 ± 12 beats per minute (bpm) which was significantly decreased by application of MF (1 μM) to 302 ± 9.9 bpm (a 9.7% decrease; p<0.05; n= 10). This response returned to baseline after washout of MF (Figure 1A and B). Changes in perfusion pressure were small and variable upon MF application, but there were no statistically significant differences across the group p= 0.84; n=15). In addition to the resting bradycardia, the PND rate was increased by MF. Measurements of the duration of specific respiratory phases reveals that MF significantly decreases Te duration (Figure 1C; p= 0.02) and significantly decreases Ttot duration (p= 0.02) which results in an increased PND rate (Figure 1D; p= 0.01; n= 10).

### Mefloquine induced bradycardia persists upon parasympathetic blockade

**Figure 2:**
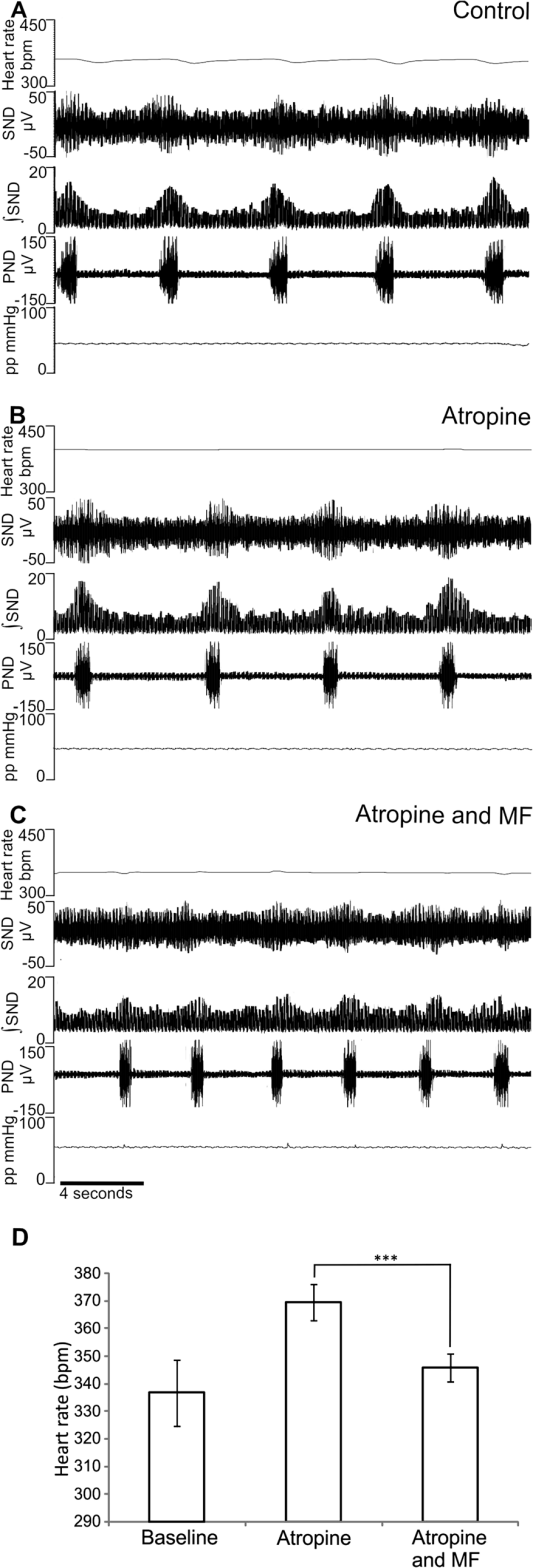
**MF induces bradycardia during parasympathetic blockade with atropine.** (**A**) Example, original traces showing baseline heart rate, sympathetic and phrenic nerve activity and perfusion pressure during control. (**B**) Addition of 1 μM atropine to the perfusate resulted in significantly increased heart rate and abolished respiratory sinus arrhythmia, indicative of parasympathetic blockade. (**C**) MF induces a bradycardia in the presence of atropine. (**D**) Average heart rate (n=4) is significantly increased from control in the presence of atropine. In the presence of atropine MF significantly decreased HR.

To determine if the decrease in baseline heart rate after MF addition was due to parasympathetic stimulation, 1 μM atropine sulphate was added to the perfusate of the rat WHBP. Consistent with parasympathetic blockade, atropine resulted in an increase in heart rate (from 336 ± 12 bpm in control to 387 ± 8 bpm; n= 4; Figure 2A and B; p= 0.01) and abolished respiratory sinus arrhythmias (Figure 2B and C). Nevertheless, in the presence of atropine MF elicited a significant decrease in heart rate from 387 ± 8 bpm with atropine alone to 351 ± 11 bpm with atropine and 1 μM MF (Figure 2C). This was an average bradycardia of 28 ± 4 bpm (n= 4; Figure 2D; p= 0.001).

### Respiratory related sympathetic activity was decreased by Mefloquine

**Figure 3:**
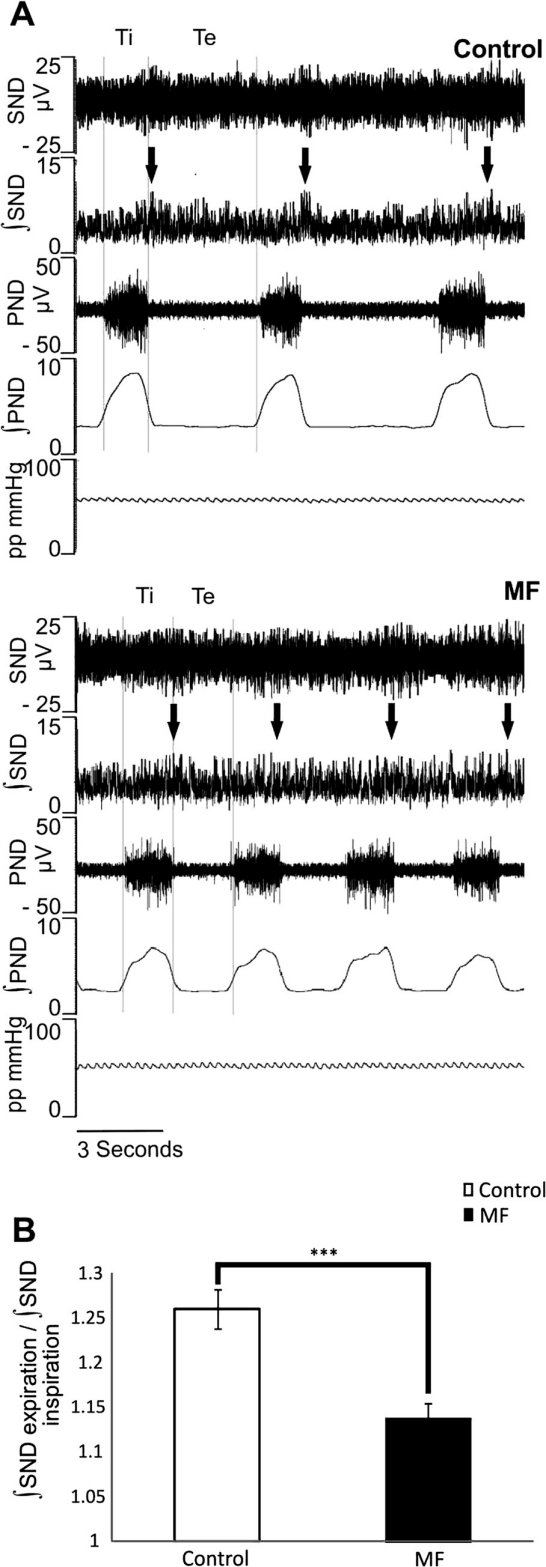
**Respiratory related sympathetic bursts are attenuated with MF.** (**A**, top panel) respiratory related sympathetic bursts are pronounced during control, arrows denote the peak in SND which occurs during the post inspiratory phase of the PND cycle. (**A**, bottom panel) reduction in the respiratory related ∫SND bursts with MF, arrows denote the same post inspiratory phase of the PND cycle that exhibited the peak ∫SND during control. (**B**) Ratio between the ∫SND during the first 500 ms of inspiration and the first 500 ms of post inspiration.

The strength of respiratory-related SND burst (which could be considered a measure of the synchrony between the two systems) was significantly decreased in MF. The ratio between the ∫SND during the first 500 ms of expiration and the ∫SND in the first 500 ms of inspiration was 1.26 ± 0.02 in control, which decreased by 9.7% with MF to 1.14 ± 0.01 (Figure 3A, B; p< 0.001; n= 6).

### Attenuation of chemoreceptor mediated sympathoexcitation with MF

**Figure 4:**
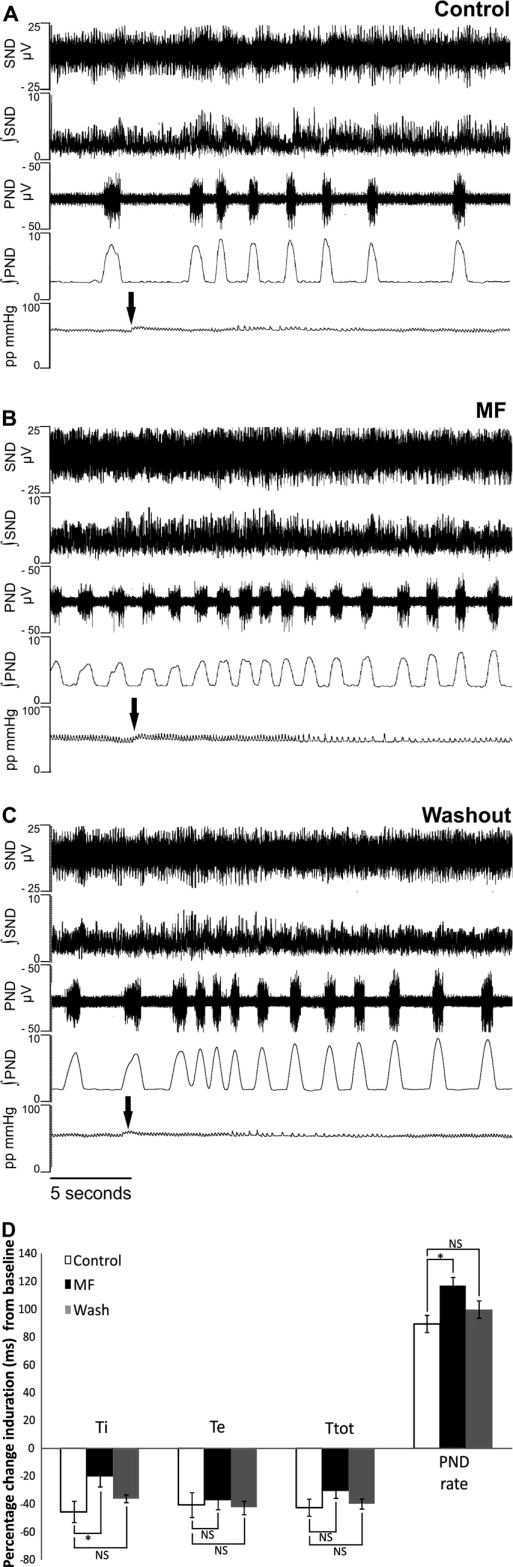
**MF augments PND discharge rate increase following chemoreceptor stimulation.** (**A**-**C**) Upon chemoreceptor stimulation (arrow) during control, MF and wash, a pronounced sympathoexcitation in ∫SND occurs and respiratory rate increases. Analysis of respiratory variables reveals no significantly different increase in Ti, Te or Ttot duration from baseline however, the PND rate significantly increased with MF compared to control and wash (**D**). Asterisks denote significance, NS; not significant.

Chemoreceptor stimulation resulted in a pronounced bradycardia, an increased PND rate and sympathoexcitation in both control and after systemic MF application (Figure 4A and B). After MF, chemoreceptor stimulation resulted in a significantly enhanced PND rate increase (Figure 4A and B). Moreover, after MF the characteristic decrease during chemo-reflex evoked tachypnoea was increased (Figure 4D; p< 0.05, n= 6).

**Figure 5:**
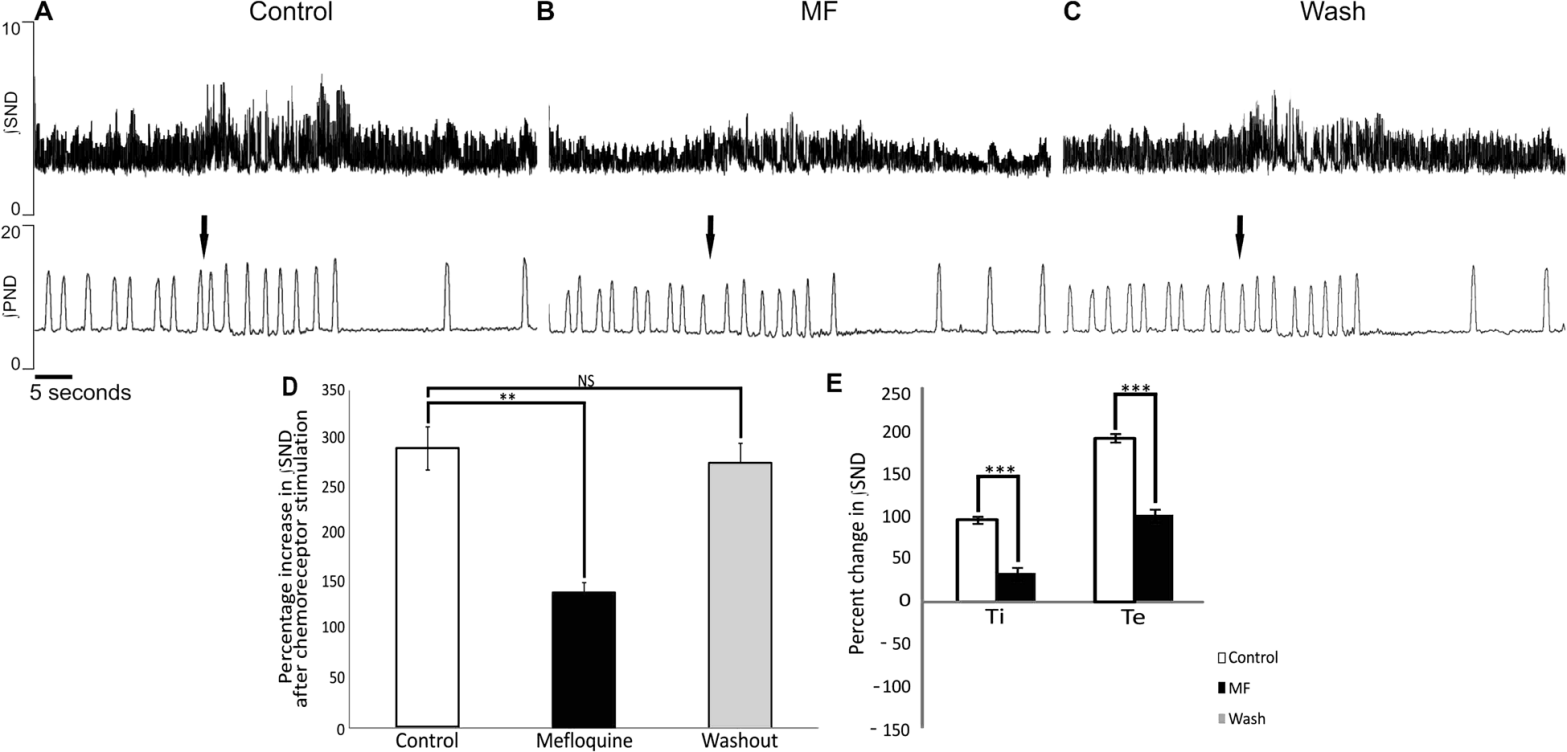
**Sympathoexcitation is attenuated with MF.** (**A**) Sympathoexcitation elicited by NaCN (arrows) during control (**A**), with MF (**B**) and after washout (**C**). Sympathoexcitation is attenuated with MF, this effect is reversed upon washout (**D**) (n = 12). Sympathoexcitation in all conditions is accompanied by increased PND rate. (**E**) Analysis of the respiratory related sympathetic bursts reveals the increase in ∫SND upon chemoreceptor stimulation is significantly attenuated during both the inspiratory and expiratory phase of the PND cycle in comparison to control. (Values are normalised from baseline values at 100%). Asterisks denote significance; NS: not significant.

Chemoreceptor reflex, activated by administration of 0.1 ml of 0.03% NaCN, also resulted in a pronounced sympathoexcitation in control, MF and recovery (Figure 5A-C). However, the sympathoexcitation was attenuated in the presence of MF (Figure 5B). Mean sympathoexcitation was reduced in the presence of MF by 51.6 ± 11.1% compared to control (p= 0.01; n= 12). After MF washout NaCN evoked sympathoexcitation recovered to control (Figure 5C and D).

Analysis of the respiratory related SND bursting during the inspiratory phase and the expiratory phase of the PND cycle revealed that with MF there was a significantly attenuated increase in ∫SND during both inspiration and expiration in comparison to control (p= 0.001; n= 10). The mean level of integrated inspiratory ∫SND during chemoreceptor stimulation in control increased by 98.1 ± 13.2% from baseline values, which was attenuated to an increase of 34.0 ± 7.0% from baseline values with MF. During expiration, the mean level of ∫SND increased by 194.13 ± 28.97% and with MF this was significantly attenuated to a 103.0 ± 16.7% increase in ∫SND upon chemoreceptor stimulation (Figure 5E).

### HR recovery time is increased by MF after chemoreceptor stimulation

**Figure 6:**
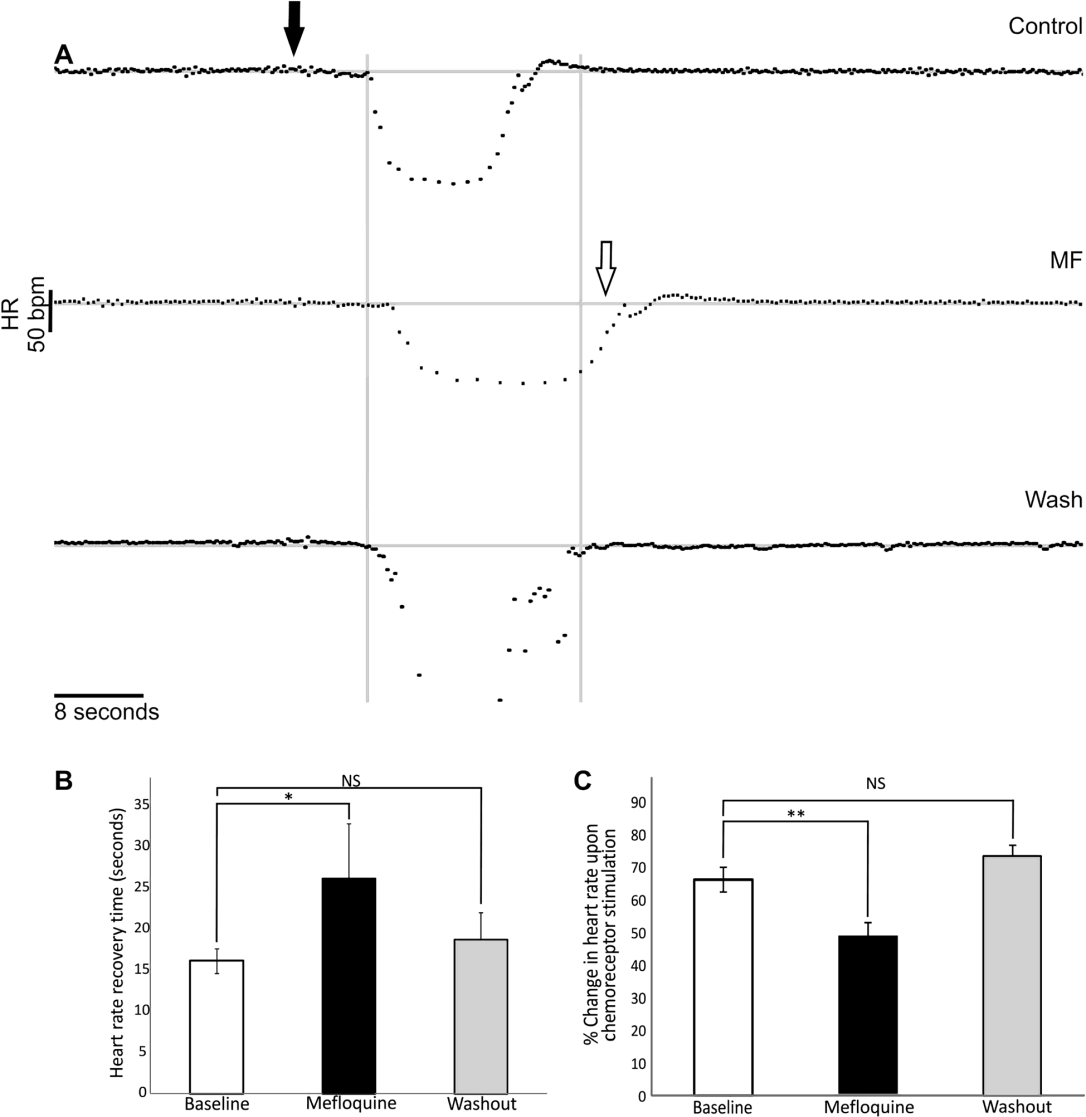
**HR recovery time following chemoreceptor stimulation is increased by MF.** (**A**) Heart rate (bpm, beats per minute) upon chemoreceptor stimulation (closed arrow) during control, MF and washout. With MF the heart rate takes longer to return to resting levels upon chemoreceptor stimulation (open arrow). (**B**) Average heart rate recovery time upon chemoreceptor stimulation is increased with MF compared to control and washout. Asterisks denote significance, NS; not significant.

Chemoreceptor stimulation elicited a pronounced bradycardia in all conditions (control, MF and wash). However, with MF, HR recovery time (time from the initial bradycardic response to HR returning to resting levels) significantly increased by 21 ± 13.7% from 16.1 ± 1.5 seconds to 26.1 ± 6.7 seconds (Figure 6; p< 0.05; n= 9). Upon MF washout, the HR return time was 18.7 ± 3.3 seconds which was not significantly different to control (Figure 6A and B). The degree of NaCN induced bradycardia was attenuated in MF. HR reduced by 66.2 ± 3.7% from baseline after NaCN administration in control. With MF, the degree of bradycardia was attenuated to a 48.9 ± 4.4% reduction (Figure 6C; p= 0.008; n= 6). Upon MF washout, the degree of bradycardia was 73.4 ± 3.3% seconds from baseline, which was not significantly different to control.

### Baroreceptor reflex sensitivity to increased perfusion pressure does not change with MF

**Figure 7:**
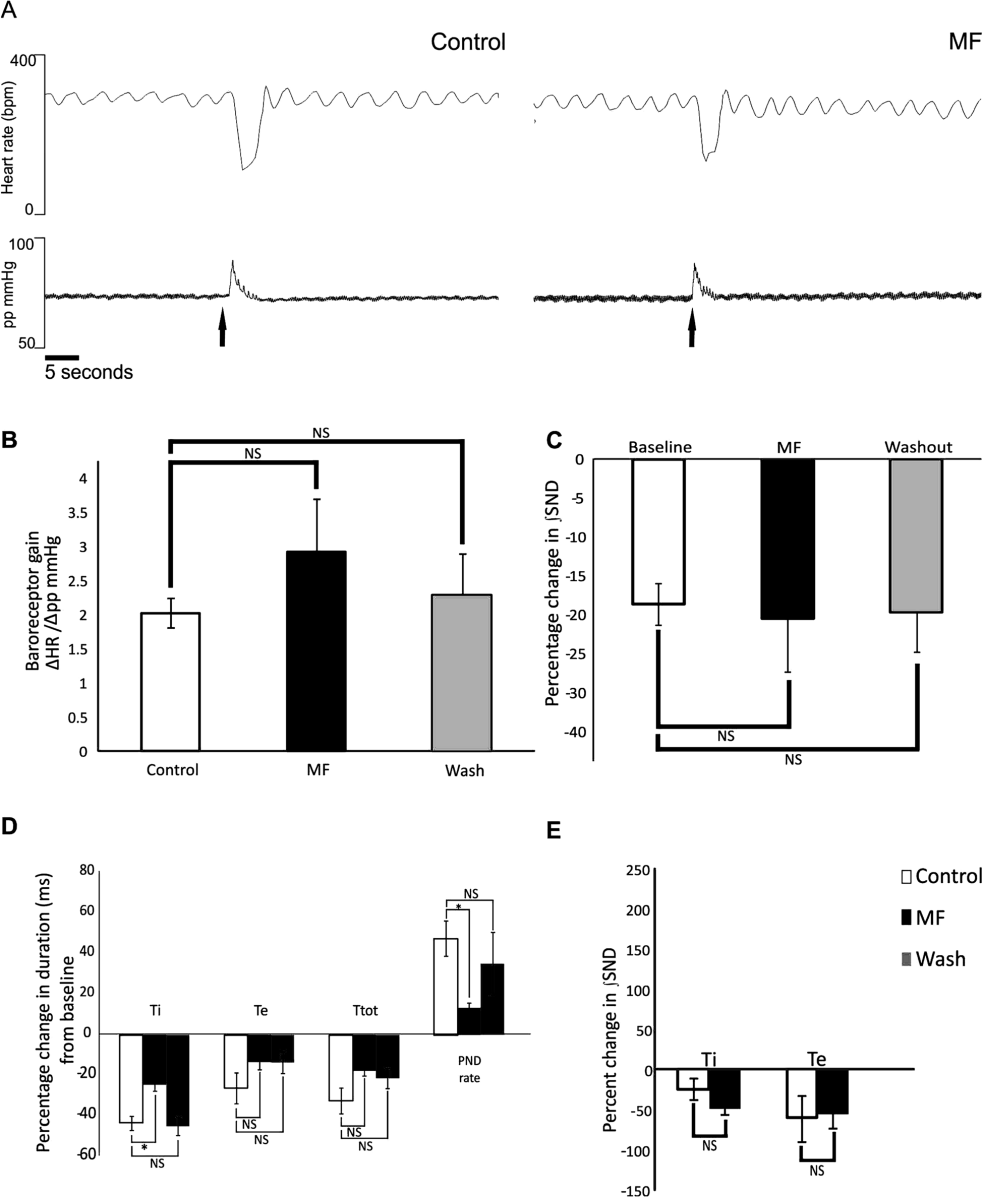
**Baroreflex sensitivity and sympathoinhibition upon baroreceptor stimulation in MF is not significantly different from control.** Stimulating the baroreceptors, with a transient increase in perfusion pressure (arrows), resulted in a reflex bradycardia (**A**). Upon application of MF to the perfusate, there was no significant change in baroreflex sensitivity (**A** and **B**) or the extent of sympathoinhibition from control (**C**). Upon baroreceptor stimulation the PND rate increase is significantly attenuated in comparison to control (**D**) due to the significantly attenuated reduction in Ti duration. (**E**) Analysis of the respiratory related sympathetic bursts in MF reveals no significant difference in the degree of sympathoinhibition in ∫SND upon baroreceptor stimulation during both the inspiratory and expiratory phase of the PND cycle in comparison to control. Asterisks denote significance, NS; not significant.

MF has no significant effect on baroreceptor reflex sensitivity to increased perfusion pressure, expressed as the change in HR rate (bpm) per 1 mmHg change in perfusion pressure, from control values of 2.27 ± 0.21 bpm × mmHg^-1^ to 2.93 ± 0.76 bpm × mmHg^-1^ (Figure 7A and B; p= 0.14; n= 6). In addition, the degree of sympathoinhibition after baroreceptor stimulation was no different with MF in comparison to control (Figure 7C; p= 0.12; n= 15). The mean inspiratory related ∫SND during baroreceptor stimulation decreased by 24.3 ± 4.3% in control and by 49.3 ± 7.5% with MF from baseline values. The mean expiratory related ∫SND decreased by 60.59 ± 4.40% in control and by 55.5 ± 7.3% with MF from baseline values. These changes from baseline values were not significantly different from control with MF (Figure 7E). Changes in respiratory parameters upon baroreceptor stimulation revealed that the average decrease in Ti duration was attenuated with MF and this also resulted in attenuation of the average PND rate increase with MF (Figure 7D; p= 0.02; n= 15) which returned to control upon washout. Therefore, an attenuated increase in respiratory parameters in response to baroreceptor stimulation in MF was observed.

## Discussion

This study shows that the application of MF to the perfusate of an arterially perfused, non-anaesthetised rat WHBP influences cardio-respiratory drive. MF resulted in a decrease in resting HR, decreased respiratory-related SND bursts, attenuation of the chemoreceptor-mediated sympathoexcitation, decreased rate of return to resting HR rate following chemoreceptor induced bradycardia and increased PND rate.

In this study MF was applied at 1 μM, which is at the lower end of the range detected in the plasma levels of patients taking MF as an anti-malarial drug [16]. The site of action of MF at this concentration may be peripheral or central, since it crosses the blood brain barrier [17]. The cardiovascular effects observed here suggest at least one route of action of MF is on central sympathetic drive, since direct recordings from the sympathetic chain revealed a reduced degree of respiratory-related sympathetic activity and a decreased response to peripheral chemoreceptor activation. Furthermore, since previous studies have excluded a direct effect of MF on the QPRS interval [4], the observation of a delayed return to resting HR after chemoreceptor activation is indicative of effects on sympathetic outflow rather than on the heart itself.

### Mefloquine induced bradycardia appears not to be mediated via parasympathetic pathways

Whilst exerting inhibitory influences on sympathetic outflow and eliciting a resting bradycardia, MF appears not to significantly influence parasympathetic outflow. Direct evidence was obtained as the MF induced bradycardia persisted during parasympathetic blockade in the presence of atropine. This was confirmed indirectly since baroreceptor reflex gain in the presence of MF was no different to control. In addition, the magnitude of chemoreceptor mediated bradycardia was attenuated in the presence of MF, suggesting that MF did not enhance vagal response to the chemoreceptor stimulus. The principle effect of MF as measured here therefore appears to be a reduced sympathetic nervous activity.

### Does Mefloquine act on Cx36 containing gap junctions in the CNS to reduce central sympathetic drive?

One potential mechanism for the MF influence on central sympathetic drive is through its well established uncoupling of Cx36-containing GJs [8]. The concentration of MF used here (1 μM) blocks 90% of the current mediated by Cx36 containing GJs when they are expressed in N2A cell lines [8] and does not affect any other connexins expressed in cardiovascular tissue [8].

Indeed, Cx36 immunoreactivity has been reported on sympathetic preganglionic neurones (SPNs) in the intermediolateral cell column (IML) of the rat spinal cord [18]. Electrophysiological studies have revealed that GJs participate in communication between SPNs [10,19]. Further, rhythmic activity recorded from the IML, the spinal nucleus containing SPNs, is reduced with the broad spectrum GJ blocker carbenoxolone (CBX) and can be abolished by MF [20]. Results from these experiments are consistent with effects of MF acting through Cx36 containing gap junctions. For example, the delayed return to resting HR following chemoreceptor stimulation suggests that the sympathetic pathways that would normally restore resting HR levels have been compromised with MF mediated blockade of Cx36 containing GJs.

However, based on the current study other sites of action of MF cannot be ruled out. For example, sympathetic outflow is influenced by inputs from the rostral ventrolateral medulla [21-23] whilst both chemoreceptor and baroreceptor afferents terminate in the nucleus of the tractus solitarius. Both these regions contain Cx36 immunoreactivity [24] which could also be sites of action for the MF in this study. Regardless of the site of action, our data show clear decreases in respiratory-related sympathetic activity in the presence of MF which suggests an uncoupling of the normal tight synchronization of respiratory and sympathetic outflows.

### Augmented respiratory drive with Mefloquine

In the current study, MF decreased the time for expiration (Te) during baseline recordings as well as more pronounced alterations in respiratory variables in response to chemoreceptor and baroreceptor activation. The potential that MF is acting on Cx36 to influence respiratory outflow is consistent with previous evidence supporting roles for GJ in respiratory control. GJs appear to be expressed throughout brainstem respiratory regions and coupled activity has been reported in these regions [9,25-27]. Indeed, in the *in situ* WHBP of the rat systemic application of GJ blockers such as heptanol, CBX and halothane reversibly decreased respiratory frequency via an increase in expiration duration [28], although these are all broad spectrum GJ blockers. Further, uncoupling brainstem GJs, using CBX, octanol or heptanol, in *in situ* WHBP increased respiratory burst frequency and decreased peak amplitude of integrated PND. In addition, PND became “bell shaped” instead of ramping with gap junctional blockade [29]. However, in contrast to the findings of [29] but in agreement with the current findings, Rodman et al., (2006) found that blocking GJs in the WHBP by CBX and octanol produced inconsistent effects, but that eupneic or gasping rhythms persisted. Similarly, using the more specific GJ blocker MF did not change the eupneic shape of the PND in the current study. However, MF evoked a decrease in Te, suggesting that Cx36 containing GJs may play a role in modulating the respiratory frequency. It may be that more than one type of GJ may be involved in respiratory rhythm generation – indeed both connexin 32 and 26 are expressed in PreBotzinger complex and chemosensitive brainstem regions [25,30]. Whilst the effects of MF in this study are consistent with a role for Cx36 GJs in cardiorespiratory control, future studies could perhaps examine this more specifically using Cx36 knockout mice.

### Clinical relevance/physiological implications of the study

MF has been a widely used anti-malarial drug with moderate to severe neurological side effects that include headache, strange or vivid dreams, dizziness, anxiety, and sleeplessness [31,32] as well as more severe neuropsychiatric adverse events such as psychosis and bipolar disorder [33]. A recent study also indicates that MF induces motor learning deficits in humans, which were associated with blockade of the known Cx36 mediated GJs in the inferior olive [34]. This current study suggests that unintended actions on autonomic and respiratory systems may also be a consideration. Indeed, there are already indications of such side effects since bradycardia and arrhythmia have been reported in some patients taking MF [4]. In particular, the reduced response to chemoreceptor challenge may have implications for MF administration prior to and during activities such as mountain climbing and diving, where chemoreceptor activation may occur. An in depth study of the effects of MF on sympathetic activity in humans, perhaps using microneurography to record sympathetic activity [35], therefore appears warranted.

## Conclusions

Application of MF to the WHBP of the rat revealed significant alterations in autonomic and respiratory activity. Further, the use of MF as an anti-malarial may require careful consideration in some populations.

## Abbreviations

aCSF: Artificial cerebrospinal fluid; Cx36: Connexin 36; ECG: Electrocardiography; GJ: Gap junction; HR: Heart rate; IML: Intermediolateral cell column; MF: Mefloquine; NaCN: Sodium cyanide; PND: Phrenic nerve discharge; SND: Sympathetic nerve discharge; SPN: Sympathetic preganglionic neurone; WHBP: Working heart brainstem preparation.

## Competing interests

The authors declare that they have no competing interests.

## Authors’ contributions

VKL completed the experimental study and the paper was written with equal contribution from VKL, JD and SAD. MD provided supervision of VKL with the WHBP and commented on the manuscript. All authors read and approved the final manuscript.
